# Quality Versus Quantity: Using Scholarly Activity to Assess Otolaryngology Residency Candidates

**DOI:** 10.1002/oto2.45

**Published:** 2023-03-02

**Authors:** Kristy J. Carlson, Jayme R. Dowdall, Katie R. Geelan‐Hansen, Dwight T. Jones

**Affiliations:** ^1^ Department of Otolaryngology‐Head and Neck Surgery, College of Medicine University of Nebraska Medical Center Omaha Nebraska USA

**Keywords:** medical students, research, residency match, scholarly activity, surgical specialty

## Abstract

Selecting qualified candidates each year for residency positions has become more difficult in recent years, due to the sharp increase in Otolaryngology applicants. Although there are objective measures that can be used to directly compare medical students during the initial screening process, most information in the application is highly subjective and/or variable across institutions. Many programs count the total posters/presentations and publications to gauge scholarship. This measure of quantity may lead to negative bias toward those who have no home program, limited time outside of academics, and/or inadequate resources to engage in volunteer research. Evaluating the quality of research may be superior to quantity. A first‐author publication is a viable proxy that demonstrates applicants have developed skills that set them apart from their peers. They likely possess non‐clinical, translatable skills including internal motivation, self‐regulation, curation of information, and task completion that map closely with qualities that make for excellent residents.

Annually Otolaryngology‐Head and Neck Surgery residency programs across the country are tasked with selecting “the best” medical students from hundreds of applicants. Most information used in the screening process is highly subjective (e.g., letters of recommendation, personal statements) or highly variable between institutions (e.g., grades, Alpha Omega Alpha) therefore, direct comparison of applicants is not feasible. Objective elements such as the US Medical License Exam (USMLE) scores and class rank are frequently used to narrow the pool of applicants for a more detailed review.^1^ The recent change in USMLE Step 1 to pass/fail removes one of the few objective measures that has been traditionally used to select students with exemplary knowledge and/or test‐taking skills.^2^ A second objective measure, USMLE Step 2, could also disappear from resident applications if the score is converted to pass/fail.

Although a holistic review of all applications is the goal for program directors, it is often not feasible due to the sheer volume of applicants that has increased substantially over the past 10+ years (Figure 1).^3^ Research activity has been a consideration for the majority of programs when deciding who to interview/rank (Figure 2).^3^ The mean number of abstracts, presentations, and publications of US MD seniors who matched in Otolaryngology has increased over time to 17.2 in 2022 (Figure 3), ranked fourth just behind plastic surgery, neurological surgery, and dermatology.^3^ Evaluating an applicant based on scholarship quantity may lead to bias against those without a home program, limited time outside of academics, and/or inadequate resources to engage in volunteer research.^4^ If this measure of volume places some students at an arbitrary disadvantage, should it be used to judge research acumen when screening applications? We believe a more reliable evaluation is the *quality* and skills demonstrated by scholarly activities, rather than quantity. Although quality is difficult to measure, a first‐author publication, regardless of field, is a viable proxy that demonstrates applicants have developed skills that set them apart from their peers. Despite the journal, the first author is known to be the person who has done the bulk of the work.^5^

**Figure 1:**
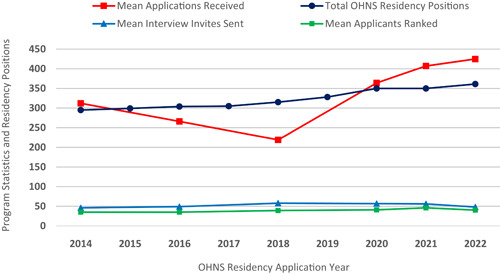
Total positions and mean applications received, interview invitations, and ranked applicants for US Otolaryngology Residency Programs 2014 to 2022. Displayed values for positions are the total number of the US applications received, interview invitations, and ranked applicants are displayed as the mean for US residency programs. The data represent a downward trend from 2014 to 2018, followed by a sharp increase in the total number of applications received. Total positions in the United States have risen over time while the mean number of interviews and ranked applicants has remained steady.

**Figure 2:**
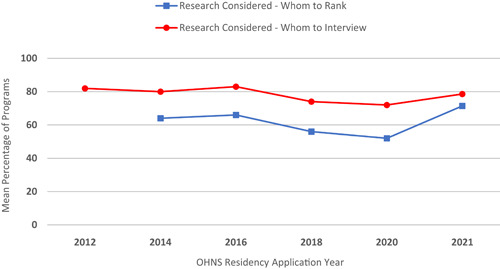
Mean percent of Otolaryngology Residency Programs consider research involvement as a deciding factor in which applicants to interview and rank from 2012 to 2021. Displayed values are the mean percentage of programs indicating research involvement was considered in decisions regarding interview invitations and the final rank list. The research was considered by more programs when deciding whom to interview than it was in ranking decisions.

**Figure 3:**
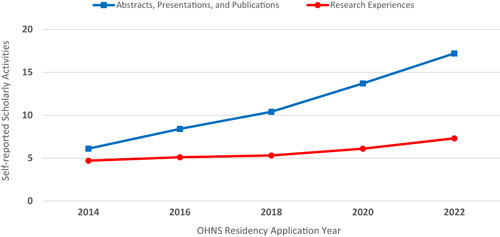
Mean self‐reported abstracts, presentations, publications, and research experiences for US MD's Matching in Otolaryngology Residency Programs 2014 to 2022. Data are based on the applicant's self‐reported abstracts, presentations, publications, and research experiences listed in the National Resident Matching Program application. This has increased substantially since 2014.

Students leading a project develop critical research and nonclinical skills necessary to complete medical training and transition to independent practice.^6^ Due to advances in technology allowing access to information on a mobile device in seconds, the development of “the art of caring,” nonanalytical aspects of medicine such as communication, leadership, team building, and creativity, has increased in importance for trainees.^7^ Common issues that occur throughout the life of a research project offer excellent teaching moments and contribute directly to the development of transferrable professional skills^8^ including the following.

## Knowledge Management

One of the challenges in selecting a project and writing research questions is managing the vast knowledge available in science. A learner must make decisions regarding the importance and quality of information assembled in a comprehensive literature search. Developing a personal system of organizing, synthesizing previous studies, and writing background is a skill that can only be learned by doing.

## Project/Time Management

Student‐led projects require planning, motivation, work/school/life integration, and perseverance. This translates directly to the ability to manage the demands of residency and has been shown to predict attrition.^8^ It also demonstrates self‐regulation and sustained interest for a student to match in a competitive residency. First authors are distinguishable from other candidates by demonstrable professional integrity. Lastly, these students have “gone the last mile” to respond to reviewer critiques and complete a project through the final and most important step: dissemination.

## Creativity/Comfort With Uncertainty

Medical students have spent the bulk of their academic careers focused on “the correct answer” to earn an exemplary grade. This laser focus results in a potential distortion of the amount of uncertainty in science.^9^ Leading research builds the capacity for self‐directed learning and exposes learners to an abyss of the unknown with endless possibilities and encourages risk‐taking and uncomfortable feelings for many students. Further, issues that arise during research require creativity and outside‐the‐box thinking.

## Exposure to Failure

Personal experience with failure creates discomfort and forces decision‐making regarding the next steps. Managing issues that arise during a project build resilience. In addition to applying new knowledge to future work, students learn to accept uncertainty and consider failure as an opportunity for growth. A first‐author publication is evidence of ownership and demonstrates the ability to work through challenges resulting in personal achievement.

## Translational Research Skills

Given the competitive environment to disseminate results at a national conference and/or reputable journal, it is critical to consider the practical application of research to avoid wasted time and effort. Students must answer the “so what” question to justify the project and identify their target audience during project planning.

## Scientific Writing Skills

The best method to improve manuscript creation is to practice. Undergraduate medical education does not typically provide opportunities to create a product designed to educate the reader, articulate importance, prioritize results, and position new information in the literature. Additional learning is realized during journal selection, rejection, and response to reviewers.

A first‐author publication is a notable accomplishment and produces deeper learning and prepares students for resident interviews designed to articulate what they have learned.^10^ Resident applicants who have achieved this are set apart from their peers because they have likely developed nonclinical, translatable skills during “the process of research.” These qualities of internal motivation, self‐regulation, curation of information, and task completion may map closely with those qualities which make for excellent residents.^4^, ^6^, ^7^, ^8^, ^9^, ^10^

## Author Contributions

**Kristy J**. **Carlson**, drafted, edited, and approved the manuscript for final submission; **Jayme R. Dowdall**, edited and approved the manuscript for final submission; **Katie R. Geelan‐Hansen**, edited and approved the manuscript for final submission; **Dwight T. Jones**, edited and approved the manuscript for final submission.

## Disclosures

### Competing interests

None.

### Funding source

None.
